# The influence of in-pregnancy smoking cessation programmes on partner quitting and women's social support mobilization: a randomized controlled trial [ISRCTN89131885]

**DOI:** 10.1186/1471-2458-5-80

**Published:** 2005-07-29

**Authors:** Paul Aveyard, Terry Lawrence, Olga Evans, KK Cheng

**Affiliations:** 1 Department of Public Health and Epidemiology University of Birmingham Birmingham B15 2TT UK

## Abstract

**Background:**

Smoking cessation interventions in pregnancy could influence a woman's social behaviour and her partner's smoking behaviour, but this has not been examined in any published randomized trials.

**Method:**

918 women smoking at booking for antenatal care were enrolled in a cluster-randomized trial of three interventions: standard care, self-help manual and enhanced stage-based counselling, or self-help manual, enhanced stage-based counselling and use of an interactive computer program. The outcomes were change in social support received by women between booking for maternity care and 30 weeks gestation and 10 days postpartum and reported cessation in the woman's partner at these times.

**Results:**

Few pregnant women's partners stopped smoking (4.1% at 30 weeks of gestation and 5.8% at 10 days postpartum) and the probability of quitting did not differ significantly by trial arm. Women's scores on the Inventory of Socially Supportive Behaviors showed a slight decline from booking to 30 weeks gestation, and a slight increase to 10 days postpartum, but these changes did not differ significantly by trial arm.

**Conclusion:**

The stage-based interventions tested in this trial aimed partly to influence women's mobilization of support and might have influenced partners' quitting, but there was no evidence that they did so. Given that women and their partners often stopped smoking together, future interventions to prevent smoking in pregnant women could encourage both partners to quit together.

## Background

There are 44 trials in the Cochrane review of interventions for smoking cessation in pregnancy[1], which show that advice and support to stop smoking doubles the quit rate. None of these trials have assessed outcomes outside women's smoking and indices of perinatal well being of the fetus and child. The authors of the Cochrane review suggest that future studies of smoking cessation in pregnancy record the effects on family functioning, meaning a basket of social and emotional outcomes.

In a comment on the Cochrane review of smoking cessation interventions in pregnancy[1], Oliver contrasts it with another Cochrane review[2], which evaluated the outcomes of social support for disadvantaged mothers on maternal and child well being[3]. Oliver makes the point that the determinants of continuing smoking throughout pregnancy include younger age[4], lower socio-economic status[5], continued psychological stress[6], low social participation, low instrumental support, and low support from a woman's partner[7], all of which is associated with nicotine dependence[8]. Given that nicotine dependence and smoking in pregnancy is embedded within socio-economic disadvantage to such a degree, we might expect that both sets of studies within the Cochrane reviews to have a similar range of broad outcomes. However, this is only true for those studies where the intervention was social support and not for those studies examining smoking cessation advice in pregnancy. Commenting particularly on the oft-cited role of cigarettes as stress-reduction agents, Oliver states "it seems irrational to try to take away a coping mechanism and not look for any social and emotional consequences such as strained family relationships" (p275)[3]. An alternative view, not discussed by Oliver, is that interventions to assist pregnant women stop smoking might actually improve family functioning if the intervention has at least some potential to do so. People who stop smoking are on average less stressed than when they were smoking[9]. This is the report of one trial of smoking cessation advice in pregnancy and its influence on two disparate secondary outcome measures that reflect family functioning. These are partners quitting and the social support given to the pregnant woman.

We found only one non-randomized intervention study that has examined the influence of smoking cessation advice for pregnant women on any of the outcomes that might constitute family functioning. Wakefield et al trained midwives to help women stop smoking[10]. Women were shown a model of a fetus *in utero *and played the recording of the change in fetus' heartbeat when a woman smoked, and women were given an explanation of this. In addition, women were given additional time with the midwife to discuss smoking cessation, and given a small booklet to take home to assist smoking cessation. Women's partners in the intervention hospital were more likely to quit compared to the partners of both a group of women in the same hospital whose smoking was monitored prior to the intervention period and to women contemporaneously observed in another hospital. The OR for partners trying to quit while the woman was pregnant in the intervention group relative to the control groups was 2.94 (1.10–7.88), representing 34.0% versus 14.9%. However, the rates of successful quitting by partners were low and not significantly different at 1.8% in the intervention group and 2.1% in the control group. Nor were there any significant differences in partners' daily cigarette consumption. At six months postpartum, there were no significant differences in attempts to quit, successful quitting, or daily cigarette consumption. Presumably, if this intervention affected the partners, it could have acted either through changing the personal interaction between the pregnant woman and her partner, or directly through sharing the self-help intervention. However, given the control group was non-randomized, any differences in partners' smoking habits could be due to inherent differences in the intervention and control groups initially, and not a result of the intervention. No currently available randomized trials have examined these issues.

We have previously reported the primary outcome at the end of pregnancy and 18 months *postpartum *and two other secondary outcomes of the trial[11,12]. Women who were still smoking at booking for antenatal care were enrolled in a randomized controlled trial to assist smoking cessation. The trial compared a standard care intervention with two different programs based on the Transtheoretical Model (TTM). We found that there were small differences between the two TTM arms. Combining the two TTM arms in the analysis (as pre-planned), the OR (95% confidence intervals (CI)) for stopping smoking at 30 of gestation weeks were 2.09 (0.90–4.85) for 10-week sustained abstinence and 2.92 (1.42–6.03) for point prevalence abstinence relative to controls. At 10 days after delivery, the OR (95%CI) for quitting were 2.81 (1.11–7.13) and 1.85 (1.00–3.41) for 10-week and point prevalence abstinence respectively. The absolute benefit of the intervention was low because only 3% or so of women managed sustained abstinence. Nevertheless, there was some evidence that the intervention benefited women's smoking, but what effect did the intervention have on women's social functioning and their partners' smoking habits? These were pre-planned secondary outcomes. The trial was not explicitly designed to influence these outcomes, but had elements of the intervention that made it plausible that it might do so and we were responding to the calls described above to report these outcomes.

## Methods

We obtained ethical approval from the relevant NHS ethical committees. The methods and main outcome of this trial have previously been reported in detail elsewhere[11,12], as have an analysis on stages of change outcomes[13], and stress in the pregnant woman[14]. Briefly, we recruited 16 of the 19 midwifery services for the West Midlands to participate in the trial. Midwives deliver antenatal care mainly in community settings, meaning general practices, rather than hospitals. About half of the available general practices were selected to participate, with only one midwife declining. Midwives were asked to attempt to recruit all women aged 16 years and over who were still smoking at booking for maternity care (about 12 weeks of gestation on average). We estimate that they recruited approximately 42% of potentially eligible smokers, described fully in the previous report[11,12]. In brief, nearly all were white, almost two thirds of women had had a baby previously, were of mean (SD) age 26.5 (5.9) years, of average net household income of £100–£200 per week, and, on average, left education aged 16 years. Women smoked on average 6 cigarettes per day at booking, but this increased to 11 cigarettes from mid-pregnancy onward[15]. The median Fagerstrom Test for Nicotine Dependence (measured at booking for maternity care) was 3, with the 10^th ^and 90^th ^percentiles being 0 and 6[16]. Three points and below represents low dependency, which might reflect a reduction in cigarette consumption that occurred in women at around the time of booking for maternity care[15]. Six points and above represents high dependency. Two thirds of women lived with partners that smoked.

### Interventions

In this pragmatic trial[17], we examined three interventions: Arm A, Controls; Arm B, Manuals; and Arm C, Computer. Midwives in each trial arm were aware that they were one of three trial arms.

#### Arm A, controls

The intervention in Arm A was intended to be standard smoking cessation advice given by midwives. Midwives in Arm A received half a day's training on the research protocol only. They were asked to deliver smoking cessation advice as they would normally do. Midwives gave women the Health Education Authority (of England) leaflet *Thinking about Stopping*. This single 21 × 30 cm sheet was folded into a 3-page leaflet and contains one section on why women should stop smoking, and five sections on how to do so.

#### Arm B, manuals

Midwives in Arms B received 2 1/2 days training. Two days covered the Transtheoretical Model, and a half a day covered the research protocol, as for Arm A midwives. Following this, midwives practiced recruiting women and using the materials and then had a half-day's reflective session on their experiences and for them to recheck details of the intervention.

At booking for maternity care, midwives gave participants in trial Arm B received a set of six 15 × 21 cm 30-page stage-based self-help manuals; "Pro-change programme for a healthy pregnancy". The set consisted of one manual for each stage of change and a further one for "recycling". These manuals explained the concepts of stage of change, helped participants to stage themselves, and contained quizzes and exercises to engage the stage-appropriate processes of change. Additionally, at each of three occasions during pregnancy booking, (generally about 12 weeks of gestation but up to 20 weeks) (named T1); 23–25 weeks (T2); and 28–30 weeks (T3), and 10 days postpartum (T4), the midwife assessed a participant's stage of change. Midwives were encouraged to discuss the use of manuals for no more than 15 minutes, such as by going through one of the self-help exercise.

#### Arm C, computer

The midwives in this arm received the same training as midwives in Arm B. The participants also received the same stage-based self-help manual intervention as Arm B and the midwife explained how to use the stage-based manuals in the same way. Additionally, these participants used a computer program installed on a laptop computer at times T1 to T4. Women worked alone without the midwife using the computer program. This consisted of questions to stage the woman, and this was followed by on-screen and audio feedback of what stage women were in and what that meant. This format was repeated for the other concepts: decisional balance, temptation, and processes of change, with strategies to use to move stage. On second and third use, women also received feedback on progress or lack of it since the last use. It took about 20 minutes to complete, and, consequently, midwives often needed an additional visit to allow women the time to complete the computer program. Following each use of the computer, the feedback was printed out and sent to the participant within one-week of the intervention.

### How could the intervention influence social support mobilization?

Neither the control intervention nor the TTM-based interventions had as a primary goal the changing of social support mobilization. Nevertheless, an intervention in smoking is an intervention on a complex biopsychosocial phenomenon, and the TTM-based intervention in particular had important elements that encouraged women to make changes in their support mobilization.

The self-help leaflet given to women in Arm A advised women to get support, but gave no advice on how this was achieved. Given that midwives offer little detailed counselling in smoking cessation to pregnant women[18], women in Arm A received very little if any intervention that could have influenced social support mobilization except that which midwives would give to all women regardless of smoking status.

Each of the TTM-based manuals gave women advice on mobilizing social support. In Precontemplation, women were advised to recognize pressure to quit from others ('nagging'), and make a plan to address this more constructively. Women were offered three pieces of advice-to acknowledge the person's concern reflected in the nagging, to tell the person that stopping smoking is a personal decision, and to remind the person that stopping smoking takes a lot of energy and stopping the nagging would allow them to consider whether stopping smoking was the right thing to do or not. In Contemplation, women were asked to imagine themselves as a non-smoker. In this guided imagining, women were told to think of the praise that family and friends might give if they stopped smoking. In Preparation, women were advised to set up a support team to help them quit (and write this down). They were asked to get the support team to congratulate them for every day without cigarettes. The Action manual emphasized the support team in the same way. In Maintenance, women were advised to anticipate the stressors they might face and make a plan of how to deal with those. They were offered a menu of items as a prompt, including 'Don't be afraid to ask for help.' The value of thinking through new ways of doing familiar tasks was also emphasized. Most of this advice described above was followed by blank areas of the book where women could write down their personal plans. Thus women in Arm B received much more advice and support to increase social support and create more positive environment than did women in Arm A.

Women in Arm C would have therefore had at least the same content as women in Arm B on social support. Plus, they would have assessed themselves on the processes of change[19]. Several processes involve social interaction. These are social liberation, which includes the creation of new social opportunities, stimulus control, which includes the control over social stimuli to smoke, and helping relationships, which means creating therapeutic relationships and enhancing the rapport in existing relationships. Women were assessed on their use of these social processes and received on-screen and subsequent written feedback on these processes to increase their use if appropriate. Additionally, women would have been assessed on the Temptation (to smoke) Scale (the complement of self-efficacy)[20], and given advice on handling social temptations, one of the dimensions of this scale, receiving on-screen and written feedback. Thus women in Arm C would have received the most intense advice addressing social support mobilization of the three groups of women.

### How could the intervention influence partner quitting?

Neither the leaflet given to the control group nor the stage-based manuals directly addressed partner quitting. The only way that these self-help interventions could therefore influence partners' smoking would be if the partners' motivation to stop was bolstered by the pregnant woman attempting to quit, or the woman shared the manual with her partner. We have no direct data on the former mechanism, which must be inferred from the quit rate data for partners presented below. However, given that the TTM-based arms increased the quit rate relative to the control group[11], this means of influencing partners is possible. Although a minority view, some men report being prepared to quit smoking to support their pregnant partners doing so[21].

The second possible means of influencing partners' quitting, lending their self-help materials, is supported by data from the follow up of these women 18 months after they had given birth. Women in Arms B and C valued their self-help materials more than did women in Arm A. For example, 10% of women in Arm A, 14% of women in B, and 22% of women in C found the self-help materials they were given either very helpful or extremely helpful. Similarly, women in the TTM arms were slightly more likely to lend their self-help materials to someone (unspecified), with 9% in A, 7% in B, and 17% in C doing so. Self-help materials are known to improve slightly the rate of quitting[22] so this may directly influence partners' quitting.

### Allocation

This was a cluster-randomized trial. The midwifery teams in each family practice were allocated by computerized minimization algorithm designed to balance the family practices across arms of the trial. The characteristics balanced by minimization were a measure of socio-economic status of the population served by the family practice (4 groups), urban/rural location (2 groups), and birth rate (3 groups).

### Outcome measures

Two outcomes were used. Women reported their partners' smoking status at booking for maternity care (approx 12 weeks of gestation) and whether their partner quit or not by 30 weeks gestation and 10 days postpartum. A previous study has shown that pregnant women's reporting of their partners' smoking habits were nearly in complete agreement with the partners' self-reports[23]. Adults in socially neutral settings, such as in response to surveys, report their own smoking accurately when checked against biochemical measures[24]. Given that women's reports of their partners' smoking agree with their partners' reports, and that partners' smoking habits are accurately reported, this implies that pregnant women report their partners' smoking habits accurately. We could not verify quitting in partners by biological measurement, but given these arguments and data, there is no reason to assume that partners' reports would be wrong, or, in particular, be more likely to be wrong in one arm rather than another. This was the first outcome.

The second outcome was the Inventory of Socially Supportive Behaviors at 30 weeks gestation and 10 days postpartum. The Inventory of Social Supportive Behaviors (ISSB) is a 40-item self-report measure that was designed to assess how often individuals received various forms of assistance during the preceding month[25]. The ISSB is an appropriate measure of support mobilization or aid provision. It measures a concept that differs from qualitative measures of support such as support satisfaction or perceived availability of social support. It asks respondents to rate how frequently certain events have happened to them in the past month. Caldwell and Reinhardt's factor structure is the most parsimonious for this scale; with clusters labelled Guidance, Emotional Support, and Tangible Support[26]. These three subscales were used as outcomes along with the global score. Guidance covered items such as 'suggested some action you should take, taught you how to do something, gave you feedback on how you were doing, gave you some information to help you understand a situation.' Emotional support covered items such as 'expressed interest and concern in you situation, was right there with you in a stressful situation, comforted you by showing you some physical affection, told you that he or she feels very close to you'. Tangible support covered items such as 'gave you under £20, provided you with a place to stay, gave you transportation, loaned you under £20.' For the component and global change in ISSB outcomes, the scores at baseline were taken from the scores at 30 weeks gestation or 10 days postpartum and this change score constituted the outcome.

In total, 918 women entered the study, of which 791 (86.1%) had a partner at booking for maternity care. Of these partners, 571 (72.2%) smoked when these women booked for maternity care. Of these 571 women, 106 (18.6%) were not followed up. The most common reasons for this were an early end to pregnancy, losing contact with the midwife, or moving house. Importantly, drop out did not differ according to arm. There was one statistically significant though fairly small difference between the women that dropped out and those that did not. Women who dropped out were less well educated, with 37% compared to 22% having no educational qualifications. However, there was no difference in drop out by baseline stage of change, cigarettes per day, Fagerstrom Test for Nicotine Dependence[16], household income, age, parity, gestation at booking, and ethnic group. Thus, drop out appeared random with respect to most characteristics.

Of the 918 women, 595 (64.8%) women had data on the change in ISSB between booking and 30 weeks gestation and 615 (70.0%) women had such data at 10 days postpartum. There was one small statistically significant difference between women with data and women in whom it was absent. Twenty percent of women with ISSB change data had no educational qualifications compared to 33% of women without ISSB change data. There were no differences in the other baseline characteristics; stage of change, cigarettes per day, Fagerstrom Test for Nicotine Dependence, household income, age, parity, gestation at booking, the proportion of women with a partner, the proportion of partners that smoked, and ethnic group. Importantly, the proportion of women with missing data was the same in each arm. Thus, drop out appeared random with respect to most characteristics.

Analysis was conducted using Multilevel Modelling for Windows (MLwiN) using random effects regression models. This accounted for the cluster randomization design. Logistic models were used for partner quitting, a binary outcome, and linear models for the change in ISSB. For both outcomes, we created both unadjusted models and adjusted models. The latter models adjusted for baseline cigarette consumption, Fagerstrom Test for Nicotine Dependence, weeks of gestation at booking, ethnic group, parity, education, income, and baseline stage of change. However, in no case did the adjusted models produce different results to the unadjusted ones and these results have been omitted.

## Results

Few pregnant women stopped smoking and also few of their partners. Of the 465 women who had smoking partners at baseline, 30 (6.5%) women had stopped smoking at 30 weeks of gestation. At the same time point, 19 (4.1%) partners had stopped at 30 weeks gestation, of which 10 (52.6%) lived with women that had quit. There was no evidence that the probability of quitting by partners differed significantly by trial arm (Table 1).

**Table 1:** The probability of partners quitting smoking by trial arm

	Arm A	Arm B		Arm C		Difference between arms
				
	%	%	OR (95%CI)	%	OR (95%CI)	χ^2^, p*
Partner quitting at 30 weeks gestation	3.3%	4.1%	1.24 (0.35–4.41)	5.2%	1.59 (0.45–5.60)	0.52, 0.77
Partner quitting at 10 days postpartum	4.8%	4.7%	0.99 (0.35–2.79)	7.9%	1.71 (0.66–4.48)	1.86, 0.40

* 2 degrees of freedom

At 10 days postpartum, 46 (9.9%) women had stopped smoking. Twenty-seven (5.8%) partners had stopped, of whom 12 (44.4%) lived with women that had stopped smoking. At 10 days postpartum, there was no evidence that the probability of quitting by partners differed by trial arm (Table 1).

The mean (SD) ISSB score at T1 was 2.0 (0.6). The means were 2.0 in all three arms, with SDs of 0.6, 0.7, and 0.6 in Arms A to C respectively. At T3, it was lower at 1.9 (0.6), but had risen after delivery to 2.1 (0.6). Table 2 shows that this pattern of small decrease to 30 weeks gestation and small increase to 10 days postpartum was the same in all three arms of the trial. There were no significant differences in change in ISSB score by trial arm. The pattern of change scores was similar for all three subscales of the ISSB.

**Table 2:** The effects of trial arm on the difference in ISSB from baseline to outcome

	Arm A	Arm B		Arm C		Difference between arms
				
	Mean	Mean	Difference B-A (95%CI)	Mean	Difference C-A (95%CI)	χ^2^, p*
Outcome at 30 weeks gestation						
ISSB combined score	-0.13	-0.09	0.04 (-0.08–0.16)	-0.08	0.05 (-0.07–0.17)	0.77, 0.68
Guidance subscale	-0.12	-0.05	0.07 (-0.06–0.20)	-0.05	0.07 (-0.06–0.20)	1.42, 0.49
Emotional support subscale	-0.15	-0.15	0.00 (-0.16–0.15)	-0.18	-0.03 (-0.18–0.12)	0.16, 0.92
Tangible support subscale	-0.01	0.02	0.03 (-0.10–0.17)	-0.06	-0.05 (-0.18–0.08)	1.71, 0.43
Outcome at 10 days postpartum						
ISSB combined score	0.09	0.10	0.01 (-0.12–0.14)	0.16	0.07 (-0.06–0.19)	1.27, 0.53
Guidance subscale	0.04	0.10	0.06 (-0.08–0.20)	0.12	0.08 (-0.06–0.22)	1.44, 0.49
Emotional support subscale	0.12	0.10	-0.02 (-0.19–0.14)	0.12	0.00 (-0.17–0.17)	0.10, 0.95
Tangible support subscale	0.10	0.08	-0.01 (-0.16–0.14)	0.15	0.05 (-0.10–0.21)	0.85, 0.65

* 2 degrees of freedom

## Discussion

Smoking is a complex bio-psychosocial phenomenon; so smoking cessation interventions usually involve quitters making changes to their social world. Hence all smoking cessation interventions have the potential to have effects on individuals other than the quitter. Smoking cessation interventions in pregnancy might be particularly able to do so because of the shared social change implied by the pregnancy for the partners involved[21]. The smoking cessation intervention in this trial had beneficial effects on women's smoking. Although the intervention did not primarily aim to influence the social world of the woman concerned and were not the major component of the intervention, the more intensive stage-based advice arms did contain considerably more advice on how to make changes than did the standard care arm. Additionally, women in the TTM-based intervention arms took home an attractive set of self-help books that they could have shared with their partner, although they were not given specific advice to do so, more than twice as many women did this in the most intensive advice arm. There was no evidence from this trial that such computerized advice and self-help literature resulted in more of the women's partners quitting smoking or women receiving more social aid provision from those around them in the intensive advice arms of the trial, however. Nevertheless, the data do emphasise that many of those women making sustained changes to their smoking behaviour were accompanied by their partners doing likewise.

There seems little scope for bias to explain these results. Approximately 42% of all potential smokers were recruited into the study. To explain these negative results by this potential bias, we would have to postulate that among the majority of non-recruits, the TTM-based intervention would have influenced social support mobilization and partner quitting favourably relative to the control intervention, but did not do so among those women recruited. Given the main reason that women were not recruited was due to midwives inactivity within the trial[11], rather than a characteristic of the women themselves, there seems to be no reason to suspect bias from this source, though it clearly cannot be excluded. Once women were recruited, the cluster randomization resulted in good balance of the characteristics of women between the arms[11], so bias from this source is unlikely also. Around 80% of women had data on change in partners' smoking habits and around 70% on change in ISSB. Clearly, if women with absent data in Arm A were different to women with absent data in Arm B or C, then bias could result. However, reassuringly, while women with fewer educational qualifications were more liable to have missing data, this effect was similar in all three arms, and bias from this source therefore also seems unlikely. It must be acknowledged that the trial had little power to exclude a worthwhile effect on partners' quitting, though it had ample power to detect small differences in social support provision.

The quit rate among pregnant women's partners was low, at 4–6%. The quit rate among partners of pregnant women was low at 4% in a nationally representative English sample[27]. Similarly, it was around 2–4% in the intervention study by Wakefield and colleagues discussed in the Introduction[10]. These data suggest that the partners of pregnant women are unlikely to stop, although this quit rate is slightly higher than the annual quit rate among all smokers (around 3%[28]). Observational data suggest that living with a partner that smokes is a major risk factor for pregnant women continuing to smoke through pregnancy[29]. Our trial data suggest that standard interventions, even those with potential to influence the partner, such as by self-help manuals used in our study, do not currently influence partners' smoking. Nevertheless, it is striking that one quarter to one third of pregnant women that stopped smoking lived with partners that also stopped. Perhaps future interventions need to test interventions that intervene on both partners in pregnancy, and not the pregnant woman alone. A qualitative study found evidence that the issue of men's smoking was not usually addressed in antenatal clinics even when the man and pregnant woman attended together. Furthermore, men reported that they would have generally welcomed support to stop smoking in that context[21].

The issue of the effect of in-pregnancy smoking cessation advice on the social functioning of women has not, to our knowledge, been addressed in any previous study. Wakschlag describe the past and current life experiences of women who continued to smoke through pregnancy[30]. Compared to women who either had never smoked or quit smoking in pregnancy, continuing smokers were more likely to exhibit problem behaviour by creating interpersonal difficulties, display problems in adaptive functioning, and engage in problematic health behaviours. Given that nicotine addiction and smoking throughout pregnancy is embedded in this constellation of problem behaviours and social disadvantage, it is unsurprising that even well-placed advice to change one's social world had negligible effect on the smokers enrolled in this trial.

## Conclusion

This self-help and midwife intervention had a small beneficial effect on women's smoking, but no benefit on either partners' smoking or on women's social functioning. More comprehensive interventions to address factors outside of the pregnant woman's smoking may have effects on family functioning and might therefore influence women's smoking more effectively also. In particular, given that a large minority of women who stopped successfully also lived with a partner who stopped, interventions that target both partners in pregnancy might be the most effective means of protecting the fetus and the child into the future.

## Abbreviations

TTM Transtheoretical Model

OR Odds ratio

CI Confidence interval

ISSB Inventory of Socially Supportive Behaviors

NHS National Health Service (of the UK)

SD Standard deviation

MLwiN Multilevel Modelling for Windows

## Competing interests

The author(s) declare that they have no competing interests.

## Authors' contributions

The trial was designed by Terry Lawrence, KK Cheng, Olga Evans, and Paul Aveyard. The study was managed by Olga Evans and Terry Lawrence. This particular data analysis was planned by Olga Evans, Paul Aveyard, and Terry Lawrence. Paul Aveyard did the data analysis and produced the initial draft of the manuscript and all other authors contributed to the revision of the manuscript.

## Pre-publication history

The pre-publication history for this paper can be accessed here:


